# A Configurable and Fully Synthesizable RTL-Based Convolutional Neural Network for Biosensor Applications

**DOI:** 10.3390/s22072459

**Published:** 2022-03-23

**Authors:** Pervesh Kumar, Huo Yingge, Imran Ali, Young-Gun Pu, Keum-Cheol Hwang, Youngoo Yang, Yeon-Jae Jung, Hyung-Ki Huh, Seok-Kee Kim, Joon-Mo Yoo, Kang-Yoon Lee

**Affiliations:** 1 Department of Electrical and Computer Engineering, Sungkyunkwan University, Suwon 16416, Korea; itspervesh@skku.edu (P.K.); yingge@skku.edu (H.Y.); imran.ali@skku.edu (I.A.); hara1015@skku.edu (Y.-G.P.); khwang@skku.edu (K.-C.H.); yang09@skku.edu (Y.Y.); yjjung@skaichips.co.kr (Y.-J.J.); gray.huh@skku.edu (H.-K.H.); seokkeekim@skku.edu (S.-K.K.); fiance2@g.skku.edu (J.-M.Y.); 2 SKAIChips, Sungkyunkwan University, Suwon 16419, Korea

**Keywords:** convolutional neural network, biosensor, diseases classification, RTL-based design

## Abstract

This paper presents a register-transistor level (RTL) based convolutional neural network (CNN) for biosensor applications. Biosensor-based diseases detection by DNA identification using biosensors is currently needed. We proposed a synthesizable RTL-based CNN architecture for this purpose. The adopted technique of parallel computation of multiplication and accumulation (MAC) approach optimizes the hardware overhead by significantly reducing the arithmetic calculation and achieves instant results. While multiplier bank sharing throughout the convolutional operation with fully connected operation significantly reduces the implementation area. The CNN model is trained in MATLAB^®^ on MNIST^®^ handwritten dataset. For validation, the image pixel array from MNIST^®^ handwritten dataset is applied on proposed RTL-based CNN architecture for biosensor applications in ModelSim^®^. The consistency is checked with multiple test samples and 92% accuracy is achieved. The proposed idea is implemented in 28 nm CMOS technology. It occupies 9.986 mm^2^ of the total area. The power requirement is 2.93 W from 1.8 V supply. The total time taken is 8.6538 ms.

## 1. Introduction

A biosensor is a device that is sensitive to biological substances and converts its concentration into an electrical signal for further processing and analysis [1]. Existing artificial intelligence (AI) biosensors have many limitations: (1) They require a large number of well-labeled data; (2) have poor flexibility, and (3) features extraction strongly depends on logic and accumulation. Due to these restrictions, the potency of traditional biosensors is limited by aspects of performance such as accuracy, timing, etc. [2,3]. During the past few years, with the promising research in deep learning, especially in CNN, these limitations can be overcome. Apart from traditional biosensor systems, CNN can meaningfully progress on extracting the different features. Therefore, based on CNN, a biosensor system can exploit the unlabeled data and the features are learned automatically by the network architecture. Hence CNN is a promising approach for biosensor applications and has also been vastly studied by existing works [4,5].

While achieving real-time performance, CNN-based techniques demand much more computation and memory resources than conventional methods. Therefore, an energy efficient CNN implementation is inevitable. Application specific integrated circuit (ASIC) and field-programmable gate arrays (FPGA) [6,7,8,9] based accelerators are promising alternates. ASIC-based study in [10,11] is proposed for the purpose of cost efficiency, energy, and throughput. Similarly, FPGA-based research [12,13,14] achieve better performance because of the parallel computation. Moreover, CNN chip implementation is categorized into two classes: (1) standard or traditional chips, and (2) neuro-chips. Standard chips are further divided into two subclasses: (I) multi-processor, and (II) sequential accelerator. In multi-processor, multiple cores are integrated for CNN operation. The purpose is to perform parallel operations for decreasing the throughput, improving the system performance and data loading by two folds. With the sequential accelerator breakthrough, the CNN algorithm on-chip integration is implementable.

The neuro-chips are built with multi-electrode arrays; a kind of integrated circuit chip that performs better because of good connectivity and fast and parallel computation. It needs low power and occupies less memory compared to traditional chips [15,16,17,18]. The research associated with neuro-chips is further classified into three main methods: fully analog, fully digital, and mixed analog/digital methods. For the mixed analog/digital [19,20], there are two operational modes; one is neuron mode, and another is synapse mode. For the neuron mode, usually, the asynchronous digital is adopted to perform the spiking. Regarding the synapse mode, most studies use analog methods to carry out compact operations such as those discussed in [21,22,23]. But there are studies that use analog/digital hybrid circuits to achieve timing synapses [24]. The digital logic foundation is adopted for on-chip structure configuration and providing corresponding algorithms. Different CNN architectures are implemented as per requirements. When using only the analog approach, mem-resistors are the key technology for implementing any functionality, keeping and tuning the resistance state according to the supply changes. Therefore, through it and resistive switching memories, it can meaningfully give the chance to achieve neural networks performance and functionalities in a fully analog manner. In [25], different functionalities of the neural network are implemented and achieved good performance in terms of small area and less power consumption. For the fully digital methods, the hardware algorithms are designed to address the functionalities and performance of the neural networks. These studies adopt software for modeling and training the neural network, get trained weights and bias values, and use these parameters for hardware implementation [26,27].

Previously, all the proposed CNN hardware implementation architectures for classification purposes used architectural parallelism and parameter reuse approaches. As a result, less memory was required or all memory on-chip was accommodated, but the drawback was moderate accuracy. In [28] and some other related studies, they also adopted parallel computation for reducing the processing time, but the trade-off was a large implementation area, nominal accuracy, and limitation of the number of layers.

The motivation of this work is to design a reconfigurable RTL-based CNN architecture for a biosensor for disease detection applications. The proposed design is fully synthesizable and technology independent. The parallel computation of MAC operation is used to reduce the arithmetic and extensive calculations. The multiplier bank is shared among all the convolutional layers and fully connected layers to reduce the implementation area.

The rest of the paper is organized as follows: Section 2 introduces the proposed top architecture of CNN for biosensor applications. The overall proposed system structure. The structures and functions of the sub-blocks are introduced in Section 3. Section 4 lists the experimental results, which includes software modeling results and hardware simulations results. Layout, mathematical results, and comparison with other works are also summarized. Finally, the paper is concluded in Section 5.

## 2. Top Architecture

**Figure 1:**
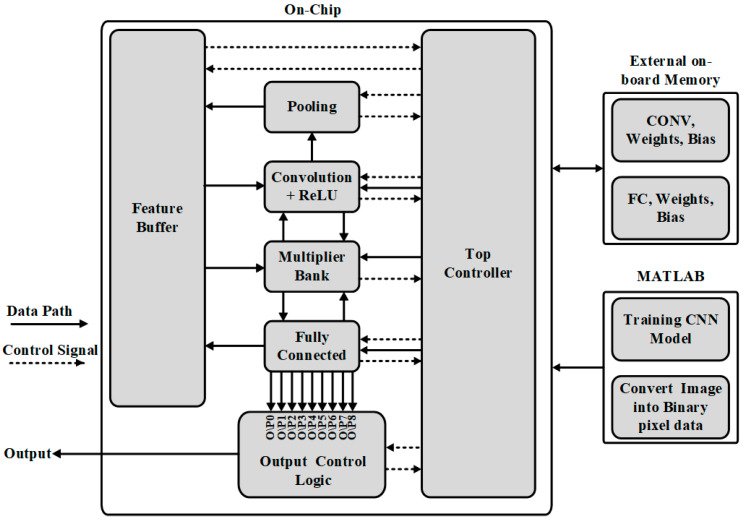
Top structure of CNN hardware implementation.

The top structure of the proposed RTL-based CNN hardware implementation is shown in Figure 1. The proposed idea is comprised of three parts: (1) CNN architecture modeling and training in MATLAB^®^; (2) External on-board memory; and (3) hardware implementation system based on RTL compiler. In MATLAB^®^, two operations are performed: (1) CNN architecture is modeled, trained, tested, and trained weights and bias data is saved in a .txt file.; (2) Converting the input feature map data into binary data, which can be recognized by hardware tools and save it into a .txt file, for further processing.

External on-board memory is a kind of multiple programmable memory. It is used for storing the trained kernels’ weights and bias values with preloaded instruction for on-chip processing. Its operation is controlled by the top controller with reading instructions and enabling signals. The interface protocol is adopted to flow the data between external on-board memory and on-chip CNN system.

In the on-chip system, the same CNN architecture is modeled. Figure 1 shows the corresponding architecture, which is comprised of several building blocks. The dotted lines represent the data path and describe the data flow direction between different sub-blocks. The solid line shows the control signal working path and indicates how sub-blocks work together. On-chip block has several sub-blocks such as the memory system, which is designed to preload and store kernel weights, bias, and feature maps data. The CNN architecture layers are convolution + ReLU layer, pooling layers and fully connected (FC) layer. The top controller controls the whole system operation, connecting the different sub-blocks and controlling the data saving on the on-chip memory or going to the next stage operation. A multiplier bank is there to perform convolution calculations. Actually, it is the main computing resource for reducing the computation and is designed to be shared among all convolution + ReLU and fully connected layers. An output control logic is designed to find the final results of the system and also for final classification results.

**Figure 2:**
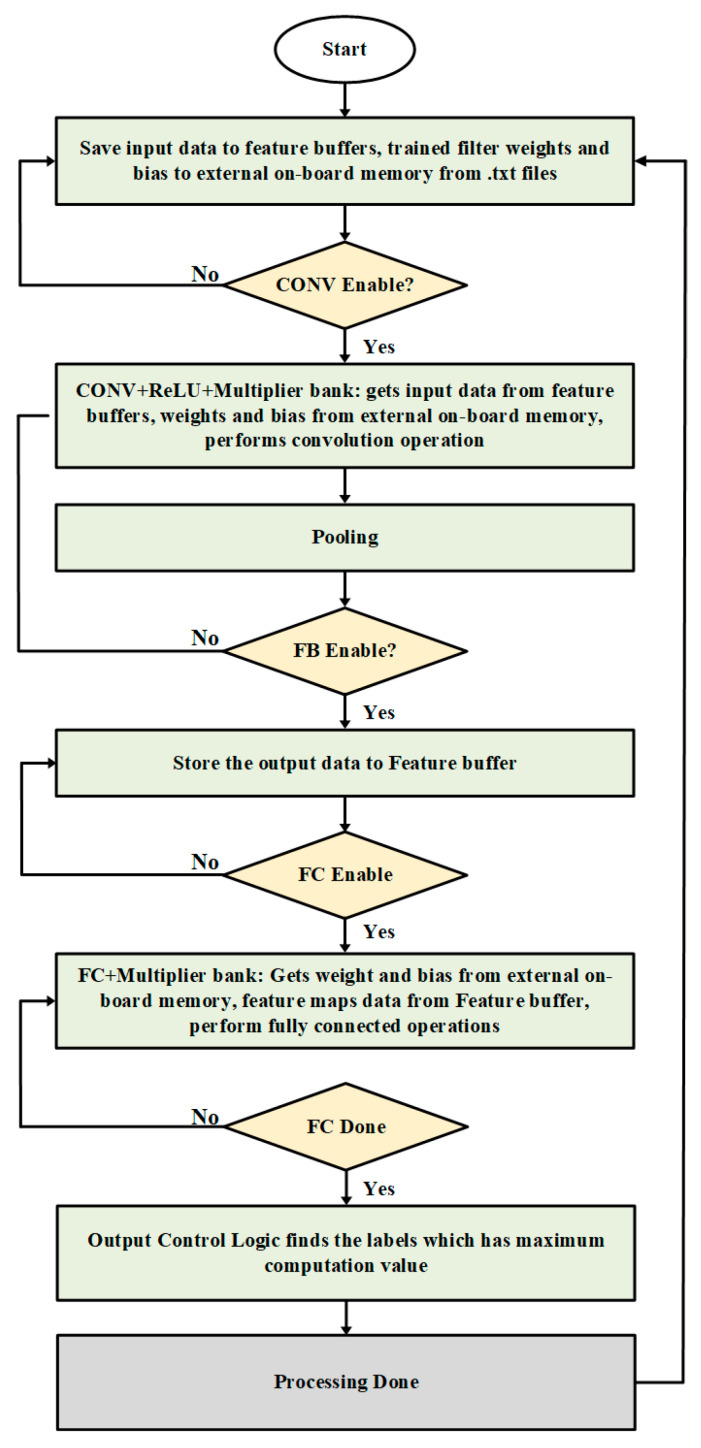
Flow chart of the proposed CNN architecture.

The on-chip system operation is described in Figure 2. As the system starts, the feature buffer saves the input data, and the external on-board memory saves trained weights and bias. Once the CONV enable signal works, the CONV + ReLU and multiplier bank get input data from the feature buffers and weight and bias values from external on-board memory, and then the convolutional operation is performed. The convolved results go to pooling layer for sampling purposes. Once all convolution and pooling iterations are done, FB enables the signal works and the output activation feature maps are saved on the on-chip feature buffers. When FC enables the signal works, the FC layer and multiplier bank obtain weights and bias from external on-board memory and feature maps from feature buffers to perform the FC operation. As the FC operation finishes, the FC done signal is generated, the output control logic finds the labels having maximum computation value, and outputs it as a class.

**Figure 3:**
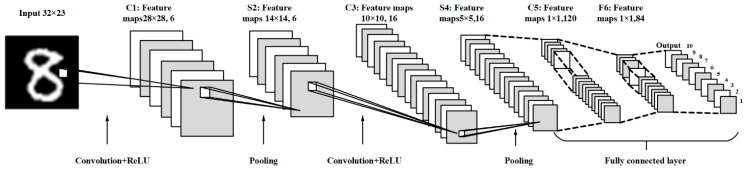
The proposed CNN model structure.

Figure 3 shows the proposed CNN architecture. It has seven layers in total, out of which there are two convolutional layers, two pooling layers, two fully connected layers, and one softMax output layer. Input feature map data dimension is 32 × 32. In C1, the input feature maps are convolved with six kernels each of size 5 × 5 with a stride of 1. It generates the six feature maps with a size of 28 × 28. Then it is handled by the activation function, such as rectified linear unit (ReLU). In S2, the size of feature maps has been sub-sampled to the half by adopting the max-pooling approach with size of 2 × 2, and the stride size is 2, to sub-sample the input feature maps to 14 × 14. In C3, 16 feature maps with 14 × 14 is convolved with a 16 kernel of size 5 × 5 to get 16 feature maps with the size of 10 × 10 and again processed with ReLU operation. The S4 max-pooling operation is operated with size 2 × 2, the stride is 2 to acheive 16 feature maps with 5 × 5. The C5 is also a convolution layer with 120 kernels each of 5 × 5 size, F6 is fully connected layer with 84 feature maps. Softmax is basically used for classification. The feature with the highest probability value is classified as an output result from handwritten digits. The CNN model is typically trained with a 32-bit floating point precision using MATLAB^®^ platform. Since the MATLAB^®^ computes parallelly, so the processing time is reduced compared to conventional C or C++ language processing approaches.

## 3. Building Blocks

### 3.1. Top Controller

The top controller is the overall controlling module of the system. Firstly, it is used to control the module sequentially with enable and done signals. When these blocks need data, the enable signal for the next operation directly starts the next module, but also disables the current module operation. Secondly, the top controller is applied for communication with external memory for reading weights and bias information. Through the interface protocol, like serial peripheral interface (SPI), the data is transferred from external on-board memory to an on-chip system. Thirdly, the top controller is also used to differentiate the read/write indicates between the contiguous blocks and manage the calculation results being saved to the memories or go to the next stage. Fourthly, it is used to control convolution + ReLU module and share the multiplier bank with the fully connected module. It controls the selection of the data information in memories, which consists of various calculation blocks to be shared multipliers or pooling blocks, and also manages the multiplier calculated values that are being sent to the convolution operation or FC operation.

### 3.2. Feature Buffers

The feature buffers, as shown in Figure 1, are used to save the output data of each sub-block. They are integrated to perform as on-chip memories. Each sub-block saves its output activation map into different on-chip memories, according to the size of the memories. These memories are built by the size of 10 k pf components, it means for each block, the capacity is 10 k bits. As for the case of the depth of the computation memory is bigger compared to this maximum size of 10 k, then another memory component is adopted which also has same size with this one.

### 3.3. Convolutional Operation

**Figure 4:**
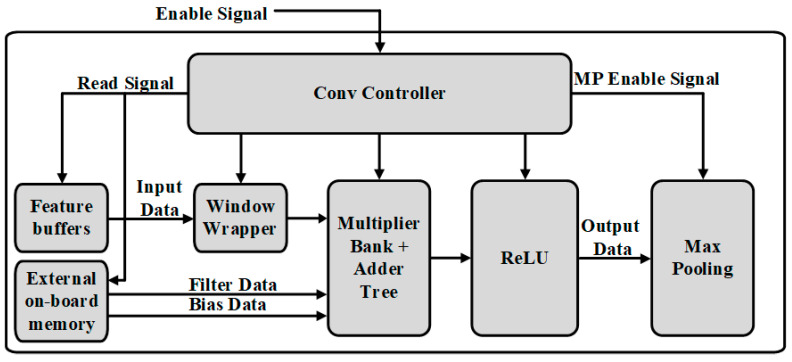
Overall architecture of the convolution layer.

The top architecture of the convolution layer is shown in Figure 4. It consists of a dedicated CONV controller, windows wrapper, multiplier bank, adder trees, and ReLU modules. The input feature maps data and kernel bias data for convolutional operation are pre-trained, after preloading, and saved in the on-chip memory. The calculation results from multiplier bank passed to adder tress for the next calculation. After computation, the results are transformed and stored into the on-chip feature buffers for next layer processing, which also has the same size with this one.

The CONV controller of convolution operation is mainly built by counters. Firstly it gets enable signal from the top controller and starts providing read signals for feature buffers and external on-board memory when convolution operation starts. Secondly, according to the kernel window size, it controls the window wrapper to select the input features maps data for partial convolution operation and slides this over all the spatial feature maps with the stride of 1. Finally, it manages the writing address to save output activation map value after ReLU processing to on-chip memories.

**Figure 5:**
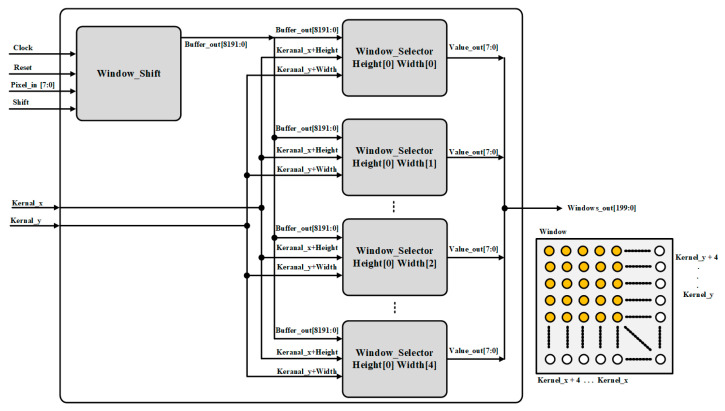
Window wrapper structure.

**Figure 6:**
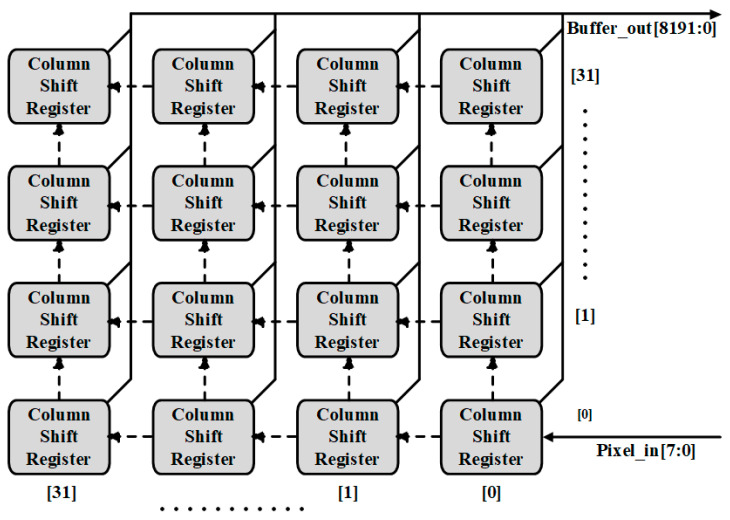
Window shift structure.

**Figure 7:**
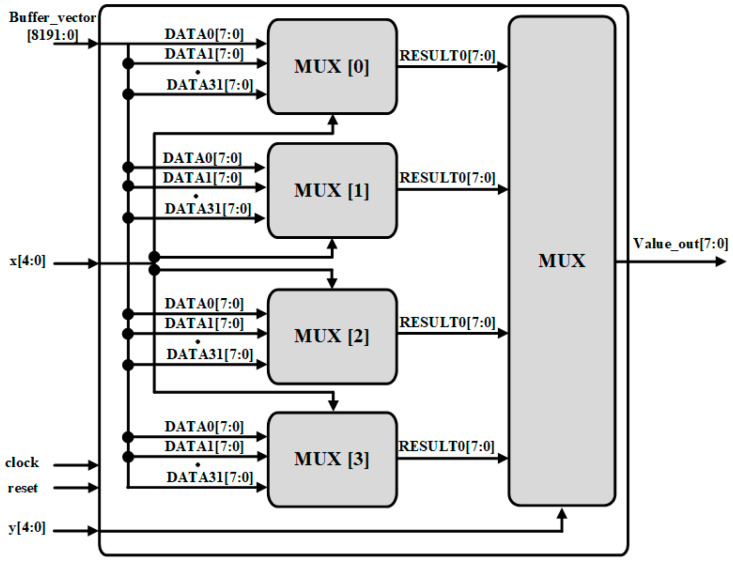
Window selector structure.

The role of the window wrapper is to select the window, as shown in Figure 5. According to the kernel size, selecting the corresponding pixels from the input feature map data. It consists of a window shifter and a window selector. The window shifter consists of shift registers, as shown in Figure 6. After obtaining feature map data from the feature buffer and the shift signal from Conv Controller, it shifts the data serially and provides it to the window selector in parallel. The window selector is consists of MUX, after getting kernel x and y coordinates from Conv Controller, it performs pixel selection, according to the kernel window size, as shown in Figure 7.

**Figure 8:**
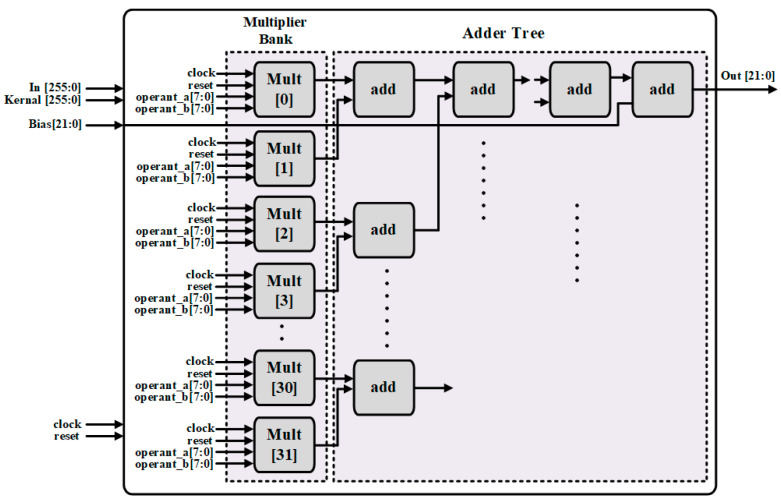
The architecture of convolutional operation.

After receiving selected pixels from window wrapper and kernel, bias values from external on-board memory, the convolution operation is performed by multiplier bank and adder tree, as shown in Figure 8. The multiplier bank consists of multipliers, and the number of multipliers is decided by the kernel window size. For example, the kernel window size is 5 × 5, so the number of multipliers should be 2^5^ = 32. Each multiplier is used to multiply 8-bit kernel values with 8-bit selected pixel in a parallel manner and provide the result to the adder tree. The adder tree accumulates the multiplier bank result, also with bias values within one kernel window. The number of addresses is decided through the multipliers, then it can be calculated as follows in (1):

(1) $$N_{adder}=\sum_{i=0}^{\log_{2}N}2^{i}+1$$

where, *N* is the number of multipliers, and *N_adder_* is the number of addresses.

**Figure 9:**
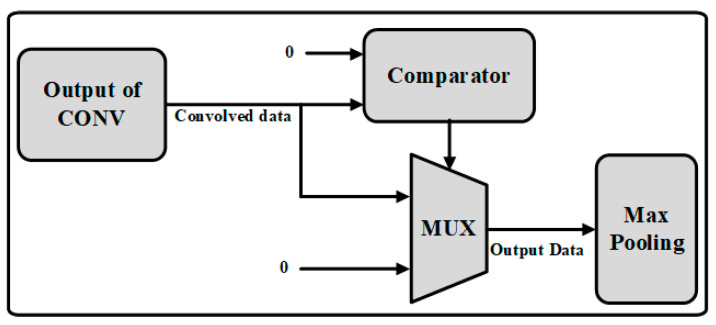
The architecture of convolutional operation.

The block diagram of the ReLU operation is given Figure 9. The adder tree in this figure consists of comparator and mux, which performs as a kernel for the assigned bit of pixel value. Basically, it converts the negative values to zero, while it leaves the positive values unchanged. Mathematically this can be given as in (2):

(2) $$f\left(x\right)=\{\begin{array}{c} 0,\;x<0\\ x,x\geq 0 \end{array}$$

where *f*(*x*) represents the output of ReLU activation function. Its output is given to the max-pooling layer directly.

### 3.4. Max-Pooling Operation

**Figure 10:**
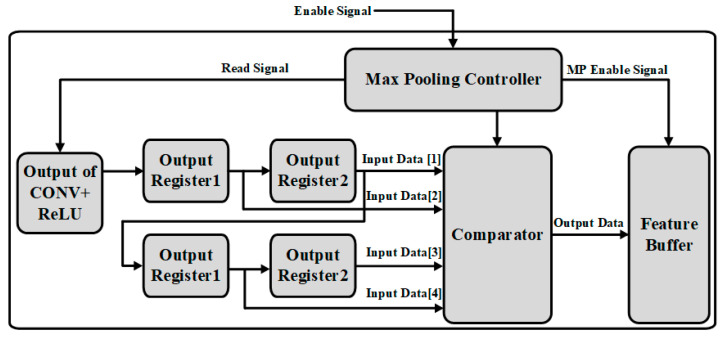
The architecture of the max-pooling operation.

The Max-pooling operation is achieved by combining the max-pooling controller with comparators. The block diagram is given in Figure 10. After receiving the enable signal from the top controller and input values from the previous layer, the max-pooling controller performs partition of the input feature maps data into a set of rectangular sub-regions with the size of 2 × 2, and the stride size is 2. The difference with window selection of convolution operation is moved without any overlapping. The comparator is used to compare the 2 × 2 sub-region values and outputs the maximum value of each sub-region. The controller also provides a read signal to the last stage for informing the start of operation and supplies a write address to save the output value to feature buffer for the next layer processing.

### 3.5. Fully Connected Operation

**Figure 11:**
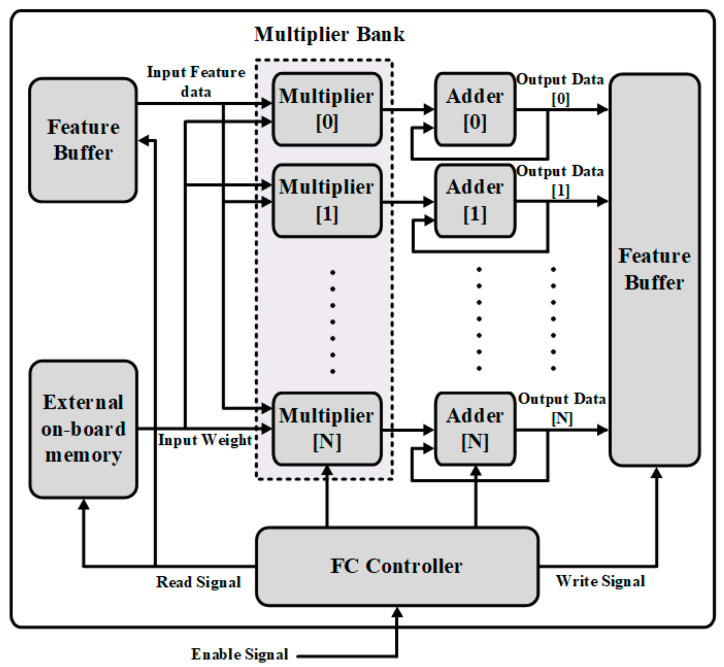
The architecture of the fully connected operation.

The function of the FC layer is the matrix multiplication. It is typically built by the the FC controller and multiplication and accumulation operations, which are similar to convolutional layers. A block diagram of fully connected operation is shown in Figure 11. After receiving enable signal from the top controller, the FC controller starts sending the read signal to the feature buffer and external on-board memory. It saves the last layer’s computation results to obtain the input feature and weight value. The parallel multiplication and accumulation computations are used to calculate the sharing feature maps data to all the rows and they are calculated together in parallel. By adopting the sharing multiplier bank with a convolutional layer, the computation is much reduced. Typically, the performance of the FC layer associates the input pitch points with output pitch points in the current layer. The function is given as in (3):

(3) $$Y_{i}=\sum_{k=0}^{W*H-1}X_{k}\times W_{i}+b_{i},N\geq i\geq 0$$

where $X_{k}$ represents the feature maps, $Y_{i}$ represents calculations results, $W_{i}$ is the weights values, and $b_{i}$ represents the bias value. *N* is the number of the output nodes.

## 4. Experimental Results

### 4.1. MATLAB^®^ Modeling and Results

**Figure 12:**
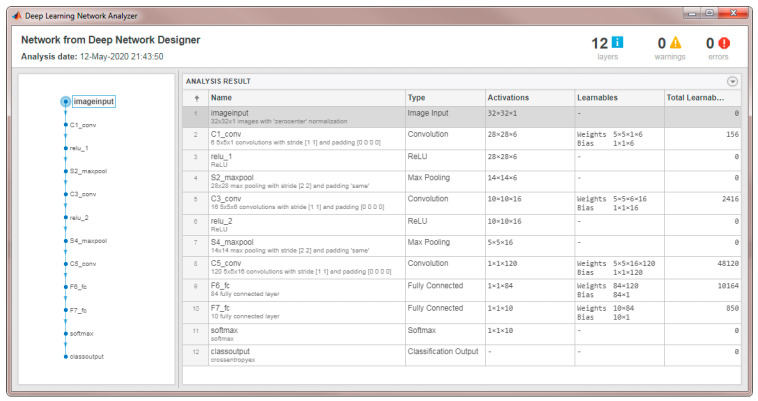
Analysis results of proposed CNN structure in MATLAB^®^.

**Figure 13:**
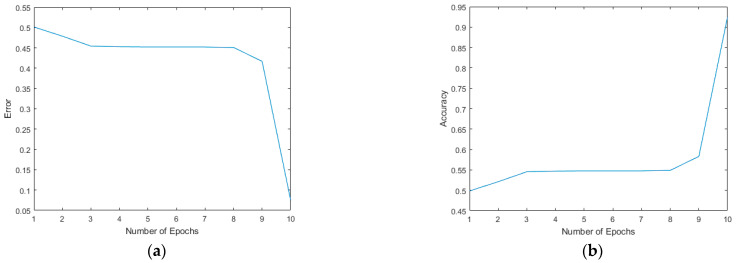
Training, testing error and accuracy of the proposed architecture. (**a**) The training error result. (**b**) The training accuracy.

**Figure 14:**
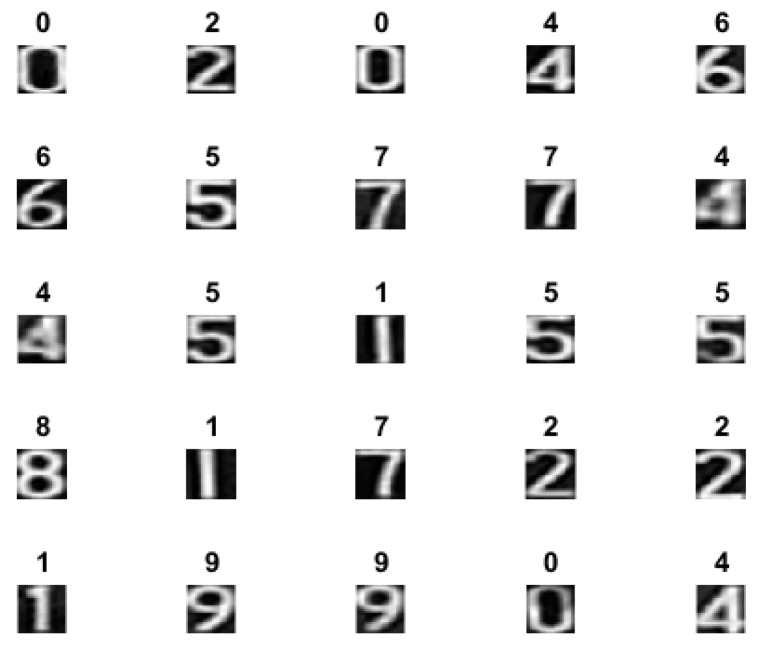
The classification result of the proposed CNN model.

The proposed CNN architecture was modeled and verified in MATLAB^®^. The model structure comprehensive analysis is given in Figure 12. It shows the layer-wise execution details, operations, operands and the total number of parameters at each layer after training. The model was trained on 60,000 images of MNIST^®^ handwritten digits [29], number of epochs were 10, with batch size of 50. The learning rate was kept 0.5. The proposed CNN model consisted of 3 convolutional layers, and 2 fully connected layers with a kernel size of 3 × 3 are used for each convolutional layer, ReLu activation function was used and max-pooling with a strip size of 2 × 2 was used. The model was tested on 1000 images from MNIST^®^ data set. Initially, a digits image is given to the model. After processing, the training error is shown in Figure 13a and the accuracy is shown in Figure 13b. We achieved 92.4977% model training accuracy. The classification results are displayed in Figure 14.

### 4.2. FPGA Implementation

**Figure 15:**
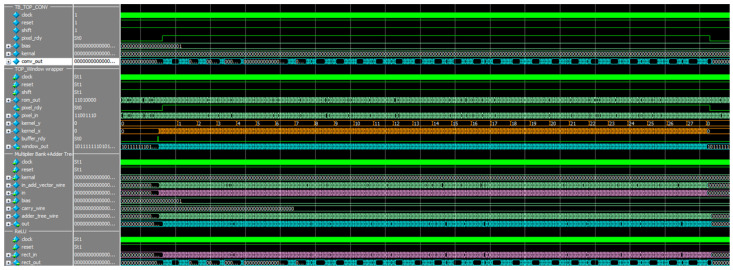
The simulation result of the top convolutional layer.

**Figure 16:**
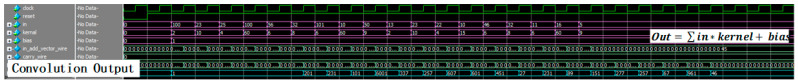
The simulation result of the convolution operation.

The simulation result of the top convolutional layer is shown in Figure 15. Image data was preloaded into image array memory; after top window selection, the selected pixels could perform the convolution operation with kernel and bias value. Figure 16 basically shows the convolution operation logic, which combined the multiplier bank and adder tree to perform operation given in Equation (4).

(4) Out=∑in×kernel+bias

where *in* is the input feature map data of convolution module, *kernel* is the corresponding weights data, and *bias* represents the system bias data.

**Figure 17:**
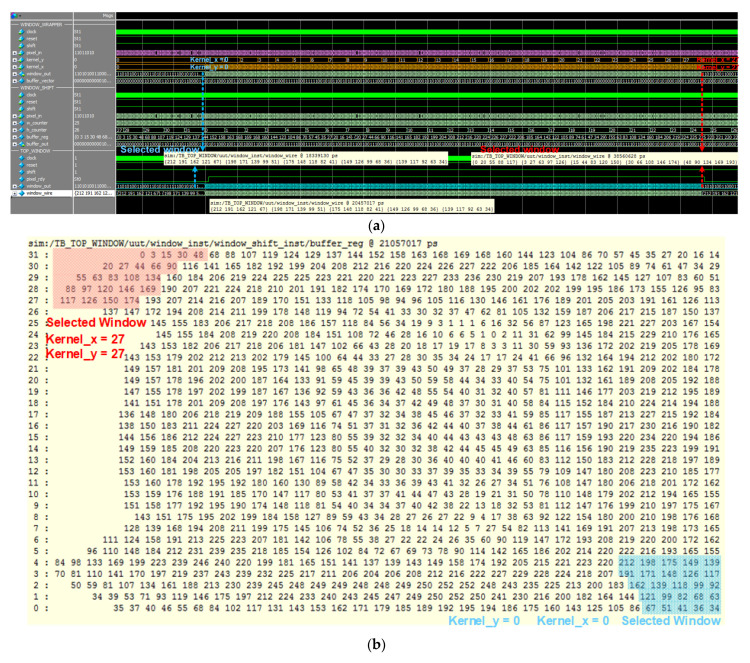
The simulation result of the max-pooling operation, where windows wrapper results are shown in (**a**) Simulation result of window wrapper, while data of one selected image is shown in (**b**) One image data with selected window.

Window wrapper simulation results are described in Figure 17, which performed the pixels selection. As Figure 17a shows, according to the kernel_x, kernel_y, the kernel window can slide around the whole image data and output the selected value which has the same size with kernel window, such as 5 × 5, Figure 17b shows the whole image data array with selected windows.

**Figure 18:**
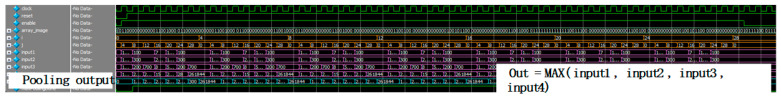
The simulation result of the max-pooling operation.

Max-pooling operations simulation results: Figure 18 shows the max-pooling operation results. When enable signal is asserted, the comparator compares the 2 × 2 sub-region values and outputs the maximum value of each sub-region. Its calculation formula is given as in (5):

(5)              Out=MAX(input1,input2,input3,input4) 

where Out represents the output value of the comparison. Input1,  input2,  input3,  input4 are the four input values of each sub-region.

**Figure 19:**
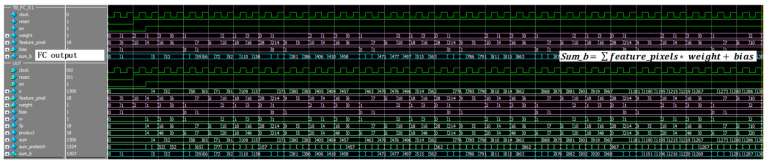
The simulation result of fully connected operation.

Fully connected operations simulation results: Figure 19 shows the FC module operation results. FC block associates input value with output value in the present module. When enable signal is high, it performs matrix multiplication. Shown as follow in (6).

(6) $$sum_{b}=\sum feature_{pixels}*\;weight+bias\;$$

where, $feature_{pixels}\;$ represents the input data of the fully connected module, *weight* represents kernel weights value, *bias* is the bias value, and $sum_{b}$ presents the output value of the fully connected module.

**Figure 20:**
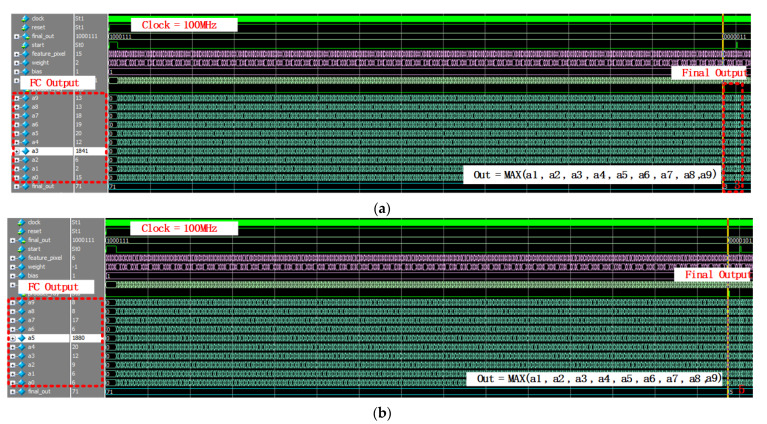

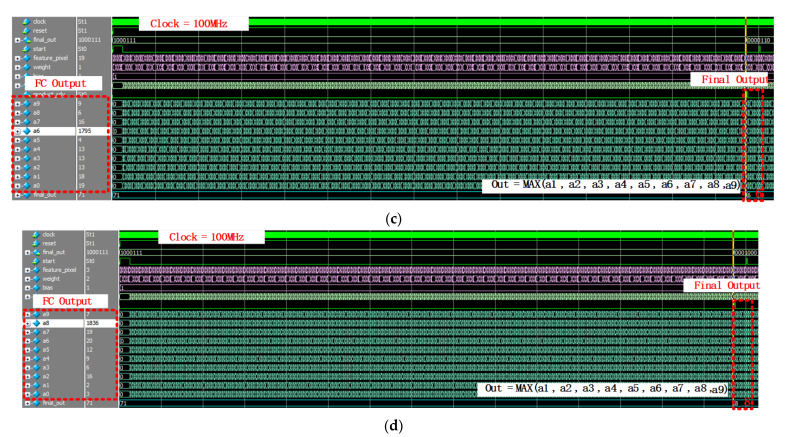
The top simulation results of the proposed CNN system. (**a**) The top simulation result of digit 3. (**b**) The top simulation result of digit 5. (**c**) The top simulation result of digit 6. (**d**) The top simulation result of digit 8.

The top simulation results of the CNN system are described in Figure 20. Compared 10 outputs of final fully connected layer, the maximum value can be found which represents final classification results. It can be achieved by (7),

(7) Y=MAX(a1,a2,a3,a4,a5,a6,a7,a8,a9) 

where *Y* is the final output of the classification results. The fully connected operation results are a1–a9. Figure 20a–d shows the classification results when the input digit is 3, 5, 6, 8  separately.

**Figure 21:**
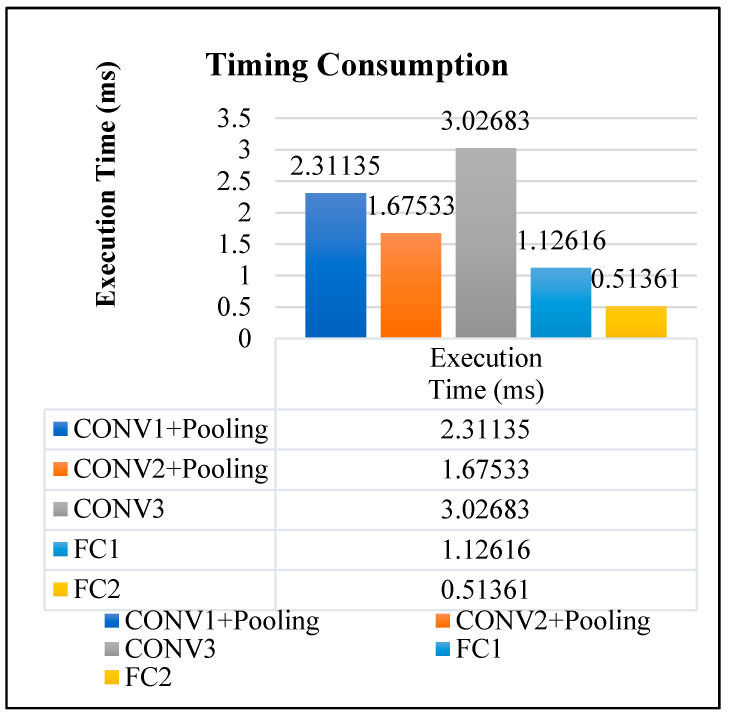
The timing consumption of different layers.

Figure 21 shows the timing consumption of different layers of the proposed CNN system. According to this table, CONV3 has the highest processing time because in this layer it has the largest number of kernels. So, the timing of loading weights and bias value to this layer and the feature map data loading time is the most costed. The total processing timing is 8.6538 ms.

**Figure 22:**
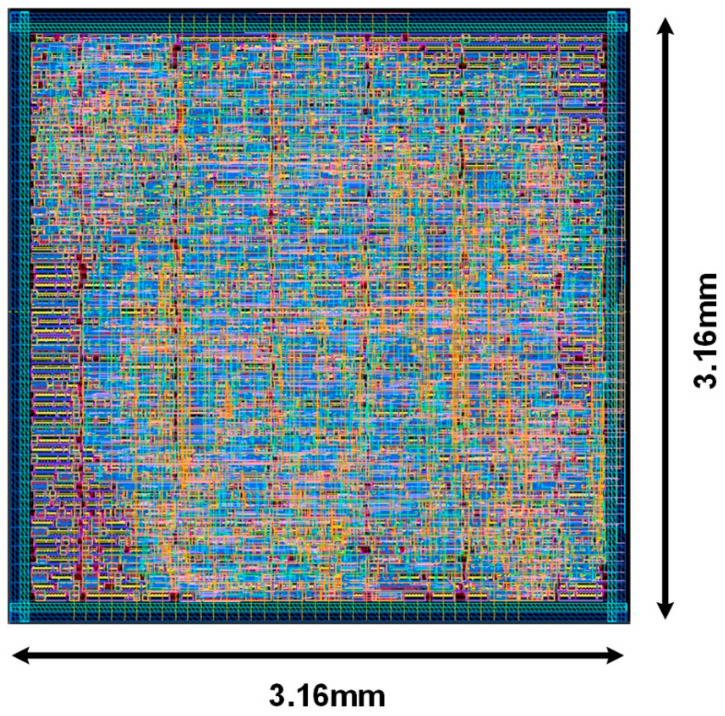
The layout of the proposed CNN system.

Figure 22 shows the layout results of the proposed CNN on the chip logic part. It is implemented on 28 nm process technology by design compiler and IC compiler. The synthesis area is 3.16 mm × 3.16 mm.

**Figure 23:**
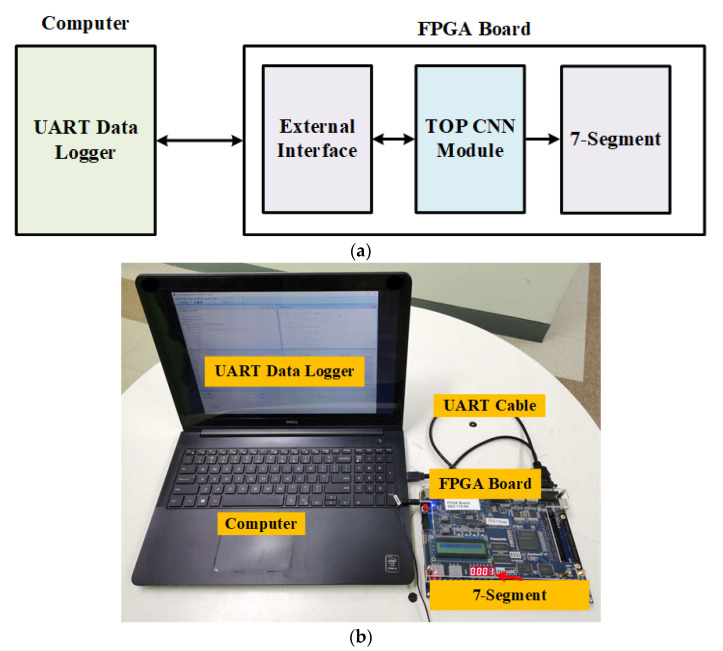
The proposed CNN system measurement setup. (**a**) The proposed CNN system measurement setup block diagram. (**b**) Measurement setup on FPGA.

The proposed CNN system is verified on the FPGA. Figure 23 shows the experimental setup for measuring the proposed CNN system. Figure 23a shows the block diagram of the measurement framework, Figure 23b shows the actual verifying lab setup situation. UART Data Logger is the software for monitoring activities of ports. It monitors data exchanged between the FPGA and an application via UART external interface, and analysis of the result for further researching. The FPGA board is connected to the computer by UART cable. After processing, the result is shown on the 7-segment on the FPGA board.

Table 1 shows the performance summaries. Compared to the other three works, [9,10,11], firstly, we can achieve the highest classification accuracy. Secondly, the on chip memory size is relatively small due to adopting the methods of sharing the multiplier bank and adder tree, especially compared to [10], which has a smaller number of layers but has a large on chip memory size. Thirdly, the power consumption is relatively low compared to the other works, which are also a fully digital-based design.

## 5. Conclusions

In the recent past, DNN and CNN have gained significant attention. This is because of its high precision and throughput. In the field of biosensors, there is still a gap in terms of the rapid detection of diseases. In this paper, we presented a synthesizable RTL-based CNN architecture for disease detection by DNA classification. The opted approach of MAC technique optimizes the hardware system by decreasing the arithmetic calculation and achieves a quick output. Multiplier bank sharing among all the convolutional layer and fully connected layer significantly reduce the implementation area.

We trained and validated the proposed RTL-based CNN model on MNIST handwritten dataset and achieved 92% accuracy. It is synthesized in 28 nm CMOS process technology and occupies 9.986 ${\mathrm{mm}}^{2}$ of the synthesis area. The drawn power is 2.93 W from 1.8 V supply. The total computation time is 8.6538 ms. Compared to the reference studies, our proposed design achieved the highest classification accuracy while maintaining less synthesis area and power consumption.

## Figures and Tables

**Table 1:** Performance comparison.

Parameter	This Work	[17]	[12]	[16]
Process (nm)	CMOS 28	CMOS 28	CMOS 65	CMOS 40
Architecture	Digital	Digital and Analog	Digital	Digital and Analog
Design Entry	RTL	-	RTL	-
Frequency (MHz)	100	300	550	204
CNN Model	6 layers	11 layers	9 layers (CNN/MLP)	-
Datasets	MNIST	CIFAR-10	MNIST	MNIST
V (V)	1.8	0.8	1	0.55–1.1
Power(W)	2.93	0.000899	0.00012	25
Accuracy (%)	92	86.05	98	98.2
On-Chip Memory	10 Kb	2676 Kb	-	-
Off-Chip Memory	40 Kb	no	-	-
Throughput (FPS)	5.33 k	-	8.6 M	1 k
Chip Area (mm^2^)	9.986	5.76	15	-

## Data Availability

Not applicable.
